# Current Evidence and Possible Future Applications of Creatine Supplementation for Older Adults

**DOI:** 10.3390/nu13030745

**Published:** 2021-02-26

**Authors:** Darren G. Candow, Scott C. Forbes, Ben Kirk, Gustavo Duque

**Affiliations:** 1 Faculty of Kinesiology and Health Studies, University of Regina, Regina, SK S4SOA2, Canada; 2 Department of Physical Education Studies, Faculty of Education, Brandon University, Brandon, MB R7A6A9, Canada; forbess@brandonu.ca; 3 Department of Medicine-Western Health, The University of Melbourne, Parkville, VIC 3021, Australia; ben.kirk@unimelb.edu.au (B.K.); Gustavo.duque@unimelb.edu.au (G.D.); 4 Australian Institute for Musculoskeletal Science (AIMSS), The University of Melbourne and Western Health, St. Albans, VIC 3201, Australia

**Keywords:** sarcopenia, osteoporosis, osteosarcopenia, frailty, cachexia

## Abstract

Sarcopenia, defined as age-related reduction in muscle mass, strength, and physical performance, is associated with other age-related health conditions such as osteoporosis, osteosarcopenia, sarcopenic obesity, physical frailty, and cachexia. From a healthy aging perspective, lifestyle interventions that may help overcome characteristics and associated comorbidities of sarcopenia are clinically important. One possible intervention is creatine supplementation (CR). Accumulating research over the past few decades shows that CR, primarily when combined with resistance training (RT), has favourable effects on aging muscle, bone and fat mass, muscle and bone strength, and tasks of physical performance in healthy older adults. However, research is very limited regarding the efficacy of CR in older adults with sarcopenia or osteoporosis and no research exists in older adults with osteosarcopenia, sarcopenic obesity, physical frailty, or cachexia. Therefore, the purpose of this narrative review is (1) to evaluate and summarize current research involving CR, with and without RT, on properties of muscle and bone in older adults and (2) to provide a rationale and justification for future research involving CR in older adults with osteosarcopenia, sarcopenic obesity, physical frailty, or cachexia.

## 1. Introduction

Sarcopenia refers to age-related reductions in muscle strength (dynapenia), muscle mass (quantity), relative strength (strength per unit of muscle mass), muscle quality (architecture and composition), and/or physical performance (i.e., tasks of functionality) [1]. Sarcopenia typically occurs in 8–13% of adults ≥60 years of age [2] and is associated with other age-related health conditions such as osteoporosis, osteosarcopenia, sarcopenic obesity, physical frailty, and cachexia. Annually, muscle mass decreases by 0.45% in men and by 0.37% in women, but these decrements climb to 0.9% for men and to 0.7% for women starting in their seventh decade [3]. The age-related decrease in muscle strength, which is a strong predictor of poor health outcomes (mobility disability, falls, fractures, and mortality) in older adults [1], occurs more rapidly (2–5 fold faster) than the reduction in lean (muscle) mass [4].

From a global health perspective, the World Health Organization established a code (ICD-10-CM; M62.84) for sarcopenia as a means for better diagnosis, assessment, and treatment of the condition. While several definitions and subcategories of sarcopenia exist, the European Working Group on Sarcopenia in Older People (EWGSOP) defines individuals with low muscle strength (as assessed by grip-strength or chair-stand test) as having probable sarcopenia; those with low muscle strength and low muscle quantity (as assessed by dual energy X-ray absorptiometry, magnetic resonance imaging and spectroscopy, computed tomography, bioelectrical impedance, creatine dilution, and/or muscle biopsy) as having confirmed sarcopenia; and those with low muscle strength, low muscle quantity, and poor physical performance (as assessed by gait speed, short physical performance battery test, timed-up-and-go test, or 400 m walk test) as having severe sarcopenia [1]. Sarcopenia is classified as primary when its etiology is age dependent whereas secondary sarcopenia is influenced by age and/or other factors such as physical inactivity and undernutrition [1]. Contributing factors to the pathophysiology of sarcopenia include changes in neuromuscular function, skeletal muscle morphology and architecture, protein kinetics, hormonal regulation, growth factors and satellite cells, vascularization, inflammation, mitochondrial function, nutrition, and physical activity [1,3,4]. From a healthy aging perspective, interventions that may help overcome characteristics and associated comorbidities of sarcopenia (i.e., osteoporosis, osteosarcopenia, sarcopenic obesity, physical frailty, and cachexia) are clinically important.

Accumulating research over the past few decades shows that creatine supplementation (CR), primarily when combined with resistance training (RT), has some favourable effects on muscle accretion and bone mineral density, bone and muscle strength, and tasks of functionality in older adults (for reviews, see Candow et al. [5], Chilibeck et al. [6], Forbes et al. [7], Gualano et al. [8], and Kreider et al. [9]). However, research is very limited regarding the efficacy of CR in older adults with sarcopenia or osteoporosis and no research exists in older adults with osteosarcopenia, sarcopenic obesity, physical frailty, or cachexia. Therefore, the purpose of this narrative review is (1) to evaluate and summarize current research involving CR, with and without RT, on properties of muscle and bone in older adults and (2) to provide a rationale and justification for future research involving CR in older adults with osteosarcopenia, sarcopenic obesity, physical frailty, or cachexia.

## 2. Creatine

Creatine is an organic acid endogenously synthesized from reactions involving the amino acids arginine, glycine, and methionine in the kidneys and liver [10]. Alternatively, creatine can be exogenously consumed from meat [9] and commercially manufactured products. The vast majority (≈95%) of creatine resides in skeletal muscle, with approximately 66% being stored as phosphocreatine (PCr) [9]. It is estimated that 2% of endogenous creatine stores are degraded daily to creatinine, a metabolic by-product of creatine metabolism [10]. For most individuals, excluding vegans and vegetarians, ≈3 g of exogenous creatine per day may help maintain creatine stores [9]. Metabolically, creatine combines with inorganic phosphate (Pi) to form PCr, which helps resynthesize and maintain adenosine triphosphate (ATP) levels [9].

## 3. Potential of Creatine Supplementation for Sarcopenia

The majority of aging research involving CR has focused on measures of muscle accretion and strength in response to RT. Studies published to date involving >600 older adults (>48 years of age) show divergent results, possibly because of methodological differences across studies (Table 1). We have previously reviewed the majority of these studies in detail elsewhere [5,8,11,12,13,14]. Most studies (*n* = 16) involved healthy older adults, whereas 4 studies involved older adults with knee osteoarthritis, osteopenia or osteoporosis, type II diabetes, or chronic obstructive pulmonary disease (COPD). The results are equivocal regarding the efficacy of CR on measures of muscle accretion and strength, with half of the studies showing greater gains from CR vs. placebo (PLA) and the other half showing similar effects between the two interventions during an RT program. Individual studies typically lack adequate statistical power to detect small changes in muscle accretion and strength from CR over time, and the responsiveness to CR in older adults may be influenced by initial resting PCr levels in different muscle regions, changes in type II muscle fibre size and quantity, and habitual dietary intake of creatine [13]. To overcome the limitations of low statistical power and high variability amongst older adult populations, three meta-analyses have been performed to determine the efficacy of CR (≥3 g/day) vs. PLA during an RT program (≥7 weeks) on measures of muscle accretion and strength [6,15,16]. Collectively, these meta-analyses showed that the combination of CR and RT augmented muscle accretion (≈1.2 kg), and upper- and lower-body strength more than PLA and RT in older adults. Mechanistically, the greater increase in muscle accretion and strength from CR may be related to its ability to influence phosphate metabolism, calcium and glycogen regulation, cellular swelling, muscle protein signaling and breakdown, myogenic transcription factors and satellite cells, growth factors (i.e., IGF-1 and myostatin), inflammation, and oxidative stress (for reviews, see Candow et al. [5], Chilibeck et al. [6], Gualano et al. [8], and Kreider et al. [9]). Upon CR cessation, the gains in muscle accretion and strength seem to persist for up to 12 weeks when RT is maintained in older adults [17].

Only three studies have determined the effects of CR and RT in older adults with different classifications of sarcopenia. Pinto et al. [37] showed that, in older adults with either probable sarcopenia (*n* = 3; skeletal muscle mass index (SMI): appendicular skeletal muscle mass/height2 <7.26 kg/m^2^ for men and <5.45 kg/m^2^ for women), sarcopenia (*n* = 1; SMI + handgrip strength <30 kg and <20 kg for women or gait speed <0.8 m/s), or severe sarcopenia (*n* = 1; SMI + handgrip strength <30 kg and <20 kg for women and gait speed <0.8 m/s), 12 weeks of CR (5 g/day) and supervised RT eliminated the probable and severe sarcopenia designations in 3 participants. However, creatine had no effect on the individual with sarcopenia. Furthermore, it is unknown whether creatine and RT reduced the level of severe sarcopenia to sarcopenia or probable sarcopenia. In seven older adults considered to be pre-sarcopenic (defined as relative skeletal muscle index >7.26 kg/m^2^ for men and >5.5 kg/m^2^ for women [39]), 8 months of CR (0.1 g/kg/day or ≈8 g/day) and supervised whole-body RT eliminated the pre-sarcopenic designation in 5 of the participants [26].

Finally, in four postmenopausal women (>60 years) who were sarcopenic (defined by appendicular lean mass, adjusted for height and weight [40]), CR (20 g/day for 5 days + 5 g/day for 23 weeks) during supervised whole-body RT (3 sets of 8–12 repetitions, 2 days per week) eliminated the sarcopenia classification in two of the women [34]. While limited by very low sample sizes, these preliminary results across studies suggest that CR (≥5 g/day) and supervised RT (>12 weeks) has some potential to mitigate sarcopenia in older adults.

Regarding physical performance (functionality), two meta-analyses of older adults demonstrated that CR in conjunction with RT resulted in greater improvements in sit-to-stand performance when compared to RT (plus PLA) alone [5,16]. These findings are of clinical relevance given that improving sit-to-stand performance may reduce the risk of falls in older adults [41].

Independent of RT, research is mixed regarding the effectiveness of CR on aging muscle, with 5 studies showing greater effects from CR vs. PLA and 5 studies showing similar effects between the two interventions (for review, see Forbes et al. [42]). While it is difficult to compare results across studies, these inconsistent findings may be related to the CR protocol and/or dosage used. The majority of studies that found beneficial effects from CR incorporated a CR loading phase (20 g/day) or used a high relative daily dosage of creatine (0.3 g/kg/day), whereas several of the studies that failed to observe beneficial effects did not use these strategies.

In summary, CR (≥3 g/day) and RT (≥7 weeks; primarily whole-body routines) can improve some measures of muscle accretion, strength, and physical performance in older adults. Independent of RT, a CR loading phase and/or high relative daily dosage of creatine (≥0.3 g/kg/day) may be required to produce some muscle benefits in older adults. It is unknown whether the combination of CR and RT provides greater fitness benefits compared to CR alone. Furthermore, the effects of CR in sarcopenic older adults is relatively unknown. No research exists regarding the efficacy of CR in older adults with inborn creatine synthesis deficiencies involving arginine–glycine amidinotransferase (AGAT), guanidinoacetate methyl transferase (GAMT), solute carrier 6 (SLC6AB), or CT1 (creatine transporter). Future research should investigate the effects of CR, with and without RT, in older clinical populations with possible musculoskeletal disorders and creatine synthesis/transporter deficiencies. 

## 4. Potential of Creatine Supplementation for Osteoporosis

**Figure 1:**
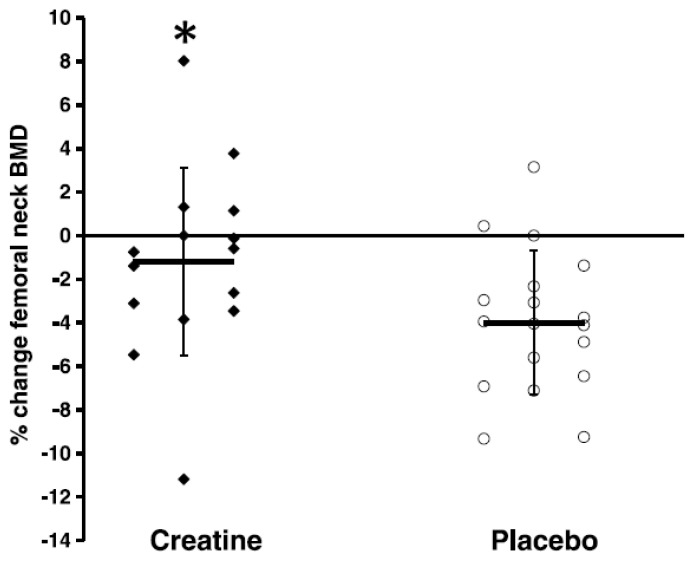
Relative changes in femoral neck bone mineral density (BMD). “Closed diamonds” represent changes for individual creatine group participants, and “open circles” represent placebo group participants. The “horizontal bars” represent the group means, and the “vertical bars” represent the SD. * Creatine participants lost significantly less BMD at the femoral neck compared with placebo participants (*p* < 0.05). (Reproduced with permission from Chilibeck et al. 2015 [28]).

Osteoporosis refers to age-related loss of bone mineral density (BMD) and architecture [43] that increases bone fragility and the risks of falls and fractures [44]. There are 8 published studies that have examined the combined effects of CR and RT on properties of bone in older adults, with only 3 of these studies showing greater effects from creatine compared to PLA (Table 2). In healthy older men, 12 weeks of CR (loading phase: 0.3 g/kg/day for 5 days; maintenance phase: 0.07 g/kg/day for an additional 79 days) and supervised whole-body RT increased upper-limb bone mineral content (assessed by dual energy X-ray absorptiometry [DXA]) compared to PLA [45]. Additional work in healthy older men showed that 10 weeks of CR (0.1 g/kg/day) and supervised whole-body RT decreased the urinary excretion of cross-linked N-telopeptides of type I collagen (indicator of bone resorption) compared to PLA [25]). Most recently, Chilibeck et al. [28] showed that CR (0.1 g/kg/day) and supervised whole-body RT for 52 weeks attenuated the rate of bone mineral loss in the femoral neck (assessed by DXA) (Figure 1) and increased femoral shaft subperiosteal width (indicator of bone bending strength) in postmenopausal women compared to PLA.

In contrast to these studies, Brose et al. [24] was unable to find a beneficial effect from 14 weeks of CR (5 g/day) and whole-body RT on serum osteocalcin (indicator of bone formation) compared to PLA in healthy older adults. Furthermore, Gualano et al. [34] found no effect from CR (loading phase: 20 g/day for 5 days; maintenance phase: 5 g/day for an additional 24 weeks) and supervised whole-body RT on changes in bone mineral (density and content; assessed by DXA) or serum concentrations of procollagen type 1 N-propeptide (P1NP; indicator of bone formation) and type 1 collagen C-telopeptide (CTX; indicator of bone resorption) compared to PLA in older women. In addition, 12 weeks of CR (5 g/day) and supervised whole-body RT had no greater effect on measures of BMD or content (assessed by DXA) compared to PLA in healthy older adults [37]. Similarly, Candow et al. [46] was unable to find greater effects from CR (0.1 g/kg/day) and 32 weeks of supervised whole-body RT on measures of bone mineral (density and content; assessed by DXA) compared to PLA in healthy older adults. Most recently, Candow et al. [27] failed to show a beneficial effect from 52 weeks of CR (0.1 g/kg/day) and supervised whole-body RT on measures of BMD or bone geometric properties (assessed by DXA and ultrasound) in older men compared to PLA.

There are only three studies that have investigated the effects of CR alone (no exercise training stimulus) on properties of aging bone. In postmenopausal women with osteopenia or osteoporosis, 24 weeks of CR (loading phase: 20 g/day for 5 days; maintenance phase: 5 g/day for an additional 23 weeks) had no effect on measures of BMD (whole-body, lumbar, total femur, and femoral neck; assessed by DXA) or serum markers of bone turnover (CTX, P1NP) compared to PLA [34]. In two additional studies involving postmenopausal women, CR (1 g/day for 52 weeks) had no effect on measures of BMD (assessed by DXA), bone microarchitecture (assessed by high-resolution peripheral quantitative computed tomography (HR-pQCT)), CTX, or P1NP compared to PLA [47]. Increasing the dosage of creatine to 3 g/day for an additional 52 weeks (104 weeks in total) also had no effect on the same bone measures in postmenopausal women. Furthermore, creatine had no effect on the number of falls or fractures experienced [48].

Collectively, the vast majority of studies show no greater effect from CR, with and without RT, on properties of bone in older adults. In the few studies that did show beneficial effects, CR was combined with supervised whole-body RT. Importantly, no study showed any detrimental effect from CR on bone mineral or geometry. The combined effects of CR and RT on reducing the risk and incidence of falls and fractures in older adults is largely unknown. Bone tissue typically takes a long time (i.e., several months) to turnover [49], especially in older adults [50]. Future research should investigate the longer-term effects (i.e., ≥2 years) of CR, with and without RT, on properties of bone mineral and geometry and risk of falls and fractures in older adults.

## 5. Potential of Creatine Supplementation for Osteosarcopenia

Osteosarcopenia is a musculoskeletal syndrome characterized by low BMD (osteopenia/osteoporosis) and muscle mass and function (sarcopenia) and is predictive of functional impairments, falls, fractures, and premature mortality in older adults [51]. Despite recent commentary proposing the use of CR to combat age-related muscle and bone loss [52,53], no randomized controlled trial (RCT) has tested the effects of creatine versus PLA (control) in osteosarcopenic adults [54]. Nevertheless, there is potential for CR, with and without RT, to be used as an upstream prevention or downstream treatment strategy for this age-related debilitating syndrome.

Creatine may directly or indirectly impact components of osteosarcopenia (muscle mass, bone density, and function) via its actions on muscle and bone metabolism. In skeletal muscle, creatine is capable of upregulating anabolic signaling pathways, increasing satellite cell number/content and growth factors, as well as downregulating markers of inflammation and oxidative stress [5,6,8,9]. Creatine’s role is not exclusive to muscle tissue, with preclinical studies showing that creatine may promote the differentiation of osteoblast cells involved in bone formation [55,56]. Despite this, in the absence of RT, findings from mechanistic studies involving CR have largely failed to translate into clinical improvements in musculoskeletal outcomes in healthy older adults. As highlighted previously, experimental trials have shown significant heterogeneity in study methodology, which likely relates to the inconsistent findings (see Table 1 and Table 2).

Of note, 2 years of CR (3 g/day) without RT did not influence bone density/micro architecture, bone turnover markers, lean mass and muscle strength/function, or falls and fractures in postmenopausal women with osteopenia [48]. However, these participants were not sarcopenic or osteosarcopenic and the RCT was not powered to detect the effects of creatine on falls and fractures, which were considerably low throughout the trial [48]. Importantly, the authors did not rule out the possible benefits of CR in conjunction with RT.

It is somewhat surprising that no RCT exists in osteosarcopenic individuals despite the well-established biomechanical and biochemical connection between muscle and bone [57]. Indeed, skeletal muscle acts as a pulley and bone as a lever during human movement and the forces applied to myofibres during RT are transmitted to bone to initiate osteocyte-induced bone formation [57], and during inactivity, the opposite occurs, leading to degeneration of both tissues. This biomechanical interaction during activity (or lack of during inactivity) occurs alongside biochemical cross-talk via hormones and other growth factors secreted by muscle and bone cells [57]. Given that lean (muscle) mass is a major predictor of BMD [58] that and osteopenia/osteoporosis increases the risk of sarcopenia (the opposite is also true) [59], it is possible that creatine’s anabolic effect on skeletal muscle may indirectly promote bone accretion and geometry. For instance, CR increases insulin-like growth factor I (IGF-1) content [60] and downregulates myostatin levels [61,62], and the former initiates osteoblastogenesis (bone formation) while the latter initiates osteoclastogenesis (bone resorption) [57]. Thus, aside from the mechanical interaction, cross-talk between muscle and bone cells represents another feasible avenue by which CR has the potential to combat osteosarcopenia. To test this hypothesis, future mechanistic studies should examine the effects of CR on hormonal factors released by the endocrine system in addition to growth factors (osteokines and myokines) secreted by muscle and bone cells. Furthermore, in order to determine the efficacy and safety of CR in older osteosarcopenic adults, future RCTs should include measures of both muscle and bone (muscle mass, strength, physical function, bone structure, and bone biomarkers) as well as clinically relevant outcomes on activities of daily living, falls, and fractures. It is also important that vital signs and adverse events are recorded in future RCTs involving creatine dosages, both of which are not consistently monitored or reported on in exercise/nutritional trials [63]. Given that adaptions in muscle mass and cortical/trabecular bone may take at least 6 months to be radiographically detected following anabolic stimuli (i.e., RT) in older osteosarcopenic adults [54], CR protocols should at least match or exceed this duration. Finally, as poor nutritional status is a risk factor for osteosarcopenia [52,53,64], the possible interaction of creatine with other essential nutrients capable of modulating muscle and bone metabolism such protein, vitamin D, and calcium [64] should be explored. Providing this information is of clinical relevance as creatine, with or without RT, may be a cost-effective strategy to treat older adults with or at risk of osteosarcopenia.

## 6. Potential of Creatine Supplementation for Sarcopenic Obesity

Sarcopenia has a negative effect on mobility, energy expenditure, and metabolic health, which subsequently increases adipose tissue accumulation (i.e., obesity [65]), especially in and around skeletal muscle [66]. Sarcopenic obesity (SO) occurs in approximately 20% of older adult populations [67] and increases the risk of cardiometabolic diseases, osteoporosis, disability, and premature mortality (for review, see Roh and Choi [68]). Similar to sarcopenia, there is no unanimous definition of SO. The WHO classifies obesity as a body mass index (BMI) ≥30 kg/m^2^. However, individuals of east Asian descent have elevated body fat% compared to non-Asians with an equivalent BMI [69]; thus, east Asian’s have a lower BMI cutoff point for obesity (≥25 kg/m^2^). Beyond BMI, body fat distribution (i.e., waist circumference) enhances the ability to predict the development of metabolic syndrome and risk of cardiovascular disease [70], with the WHO indicating cutoff values of ≥102 cm for men and ≥88 cm for women, which differ for Asian populations [71,72]. Further, the American Association of Clinical Endocrinology [73] classifies obesity using body fat thresholds of >25% in men and >35% in women. Due to the lack of a universally accepted definition, the prevalence of SO varies. For example, in a prospective study of older adults (*n* = 4652; >60 years of age), the prevalence of sarcopenic obesity was 18.1% in women and 42.9% in men [74].

A recent systematic review and meta-analysis of 19 studies involving older adults (*n* = 609 participants; ≥50 years of age) showed that the combination of CR and RT resulted in a greater reduction in fat mass (0.5 kg, *p* = 0.13) and body fat% (0.55%, *p* = 0.04) compared to PLA and RT [75]. Mechanistically, creatine appears to also influence adipose tissue biology. In multiple adipogenic cell culture models, creatine attenuated the accumulation of cytoplasmic triglycerides in a dose-dependent manner through inhibition of phosphatidylinositol 3-kinase activation [76]. There is also evidence that creatine can alter whole-body energetics and expenditure [77]. In rodents, diminishing creatine content impaired thermal homeostasis [78] and deletion of glycine amidinotransferase (the rate limiting enzyme of creatine synthesis) attenuated creatine content in brown adipose and impaired thermoregulation [77,79], which subsequently attenuated the capacity to activate diet-induced thermogenesis, resulting in increased adiposity [79]. Furthermore, global creatine transporter (Slc6a8) knockout mice presented with greater body fat stores compared to controls [80] possibly due to lower whole-body energy expenditure, a decrease in oxidative metabolism in beige and brown adipose tissue, and an increase in feed efficiency [81].

Collectively, CR and RT appear to be an effective intervention for decreasing body fat% in older adults. However, the effects of CR alone on adipose tissue biology in older adults are unknown. Furthermore, it remains to be determined whether CR, with and without RT or other exercise-training modalities (i.e., aerobic), can overcome SO. Based on the potential interaction of muscle and fat tissue and mechanistic actions of creatine on adipogenesis and whole-body energy expenditure, future research is warranted and may be of clinical importance for older adults.

## 7. Potential of Creatine Supplementation for Physical Frailty

Frailty is defined as a syndrome of physiological decline in later life, characterized by vulnerability to adverse health outcomes (i.e., hospitalization, falls, social isolation, and reduced quality of life (QoL)) [82,83,84]. Frailty is commonly defined according to the phenotype of physical frailty proposed by Fried et al. [82], which consists of weakness, slowness, low levels of physical activity, shrinking, and exhaustion, with one or two criteria indicating a prefrailty stage and three or more marking frailty. A recent study in a combined cohort of 8804 Australian adults aged ≥65 years (women 86%, median age 80 years) found that, while 21% of participants were frail, a staggering 48% were prefrail [85]. With the aging population and growing incidence of prefrail older adults progressing to frailty every year (at a rate of 11%), the incidence of adverse health outcomes represents a substantial burden on total healthcare costs worldwide.

Due to the predominantly musculoskeletal and physical components of the frailty phenotype, there is an unavoidable overlap between sarcopenia, osteosarcopenia, and physical frailty [86]. Regarding their common pathophysiology, these conditions share immune, endocrine, and inflammatory mechanisms, which could be targeted via nonpharmacological interventions such as exercise and nutrition (i.e., creatine) [64,87]. However, research is very limited regarding CR and physical frailty. Although some studies testing CR have involved older adults that could fulfil the clinical phenotype, those participants are usually labelled as healthy or non-sarcopenic. In the only clinical trial to directly assess the effects of CR in mildly frail older adults (defined as those with limited dependence on others for instrumental activities of daily living according to the Canadian Study of Health and Aging clinical frailty scale [88], Collins et al. [89] found no additive effect from CR (5 g/day) to whey protein (20 g) and RT (14 weeks) on measures of muscle strength and functionality compared to whey protein and training alone. However, the small sample size (n = 16) and lack of PLA and non-training control group limits the clinical application of these preliminary findings. In addition to the effects of CR on muscle and bone as previously described, there is evidence that creatine may have beneficial effects on the other components of the frailty syndrome (summarized in [90]). Creatine exhibits an anti-inflammatory effect via regulation of the cyclo-oxygenase pathway and reduction of serum levels of inflammatory cytokines (i.e., tumor necrosis factor-α (TNF-α) and IL-6), which have been associated with sarcopenia, osteoporosis, and frailty [91,92]. The small number of studies that have examined the efficacy of CR on immune system response have shown an alteration in soluble mediator production and expression of molecules involved in recognizing infections, specifically toll-like receptors. Creatine has also been proposed to be neuroprotective, an effect that could have a potential role in the treatment of the neuromuscular components of frailty [93]. Finally, creatine may act as an antioxidant, which would also benefit frail older adults susceptible to increased oxidative stress and damage [94].

In summary, despite the preclinical and clinical evidence demonstrating an effect from creatine on multiple pathophysiological mechanisms associated with frailty, no RCT has been performed examining the effects of CR (alone or in combination with exercise) in frail older adults. A major challenge in this line of research relates to the identification of frailty, which could lead to significant variability across studies. In the case of physical frailty, we propose that the adoption of Fried’s criteria be used to facilitate and identify the condition through a well-accepted phenotype in which the quantification of any therapeutic effect(s) from CR can be made.

## 8. Potential of Creatine Supplementation for Cachexia

Cachexia can be defined as a tissue loss syndrome that involves severe weight loss and muscle wasting [95]. Cachexia is usually secondary to conditions such as cancer, COPD, chronic kidney disease, and heart failure, and therefore, therapeutic interventions should involve not only the prevention of muscle wasting but also appropriate treatment of the secondary cause.

Muscle loss in cachexia is due to both reduced protein synthesis and increased catabolism (proteolysis) due to multiple factors including reduced oral intake, high levels of inflammation, tumor-mediated effects, low physical activity, and endocrine and metabolic disturbances [96]. Some studies have demonstrated that CR can have an effect on the majority of these mechanisms; however, PLA-controlled studies have shown mixed results. Most of the studies involving CR and cachexia have been performed in cancer patients (summarized in Table 3). Overall, CR failed to produce significant effects on muscle accretion, muscle performance, or functionality. However, creatine had no detrimental effect on muscle, bone, performance, or functionality and no major adverse events were reported from those taking creatine. The inconsistent findings across studies are possibly explained by low sample sizes, multifactorial nature of the condition, deleterious effect of chemotherapy on muscle, lack of exercise intervention, length and dosage of creatine used, and heterogeneity of secondary causes of cachexia

There may be potential for cachexia patients to experience some benefits from CR and RT. For example, some subsets of cancer are characterized by high rates of weight and muscle loss (i.e., head and neck, pancreatic, lung, colorectal, and gastric cancer) [97], which may be counteracted by creatine. Additionally, CR may also be beneficial for older adults with cachexia and/or cancer undergoing treatments that negatively affect muscle and bone mass, performance, and function (i.e., androgen deprivation therapies).

In summary, cachexia is a debilitating condition associated with multiple chronic diseases, especially cancer. Creatine has the potential to target several of the mechanisms associated with cachexia; however, research investigating the effects of creatine and cachexia is very limited. Future large-scale RCT’s examining the effects of creatine, with and without exercise and pharmacological therapies, are warranted and needed.

## 9. Conclusions

**Figure 2:**
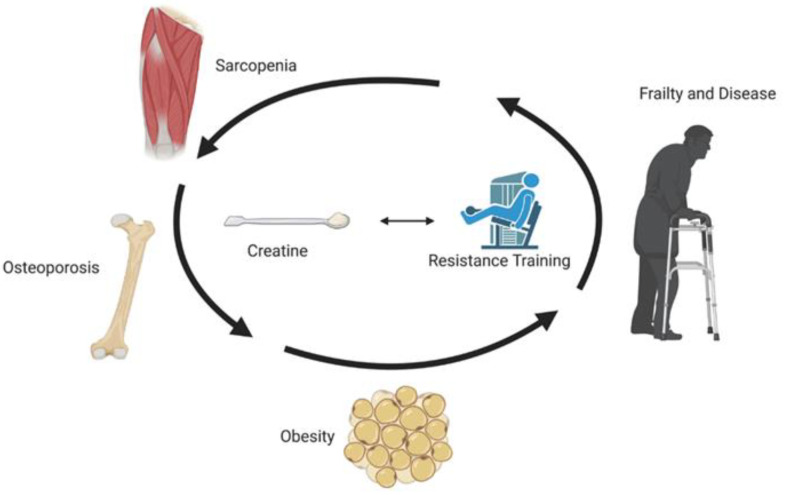
Potential effect of creatine, with and without resistance training.

Sarcopenia refers to age-related reduction in muscle mass, strength, and/or physical performance and has a negative effect on the ability to perform activities of daily living and overall quality of life. Comorbidities associated with sarcopenia include osteoporosis, osteosarcopenia, sarcopenic obesity, physical frailty, and cachexia. As a possible countermeasure to sarcopenia and its age-related co-morbidities, CR (especially when combined with RT) has some favourable effects on aging muscle, bone and fat mass, muscle and bone strength, and physical performance, primarily in healthy populations (Figure 2). Independent of RT, a CR loading phase and/or high relative daily dosage of creatine (≥0.3 g/kg) may be required to produce some muscle benefits in older adults. CR (independent of resistance training) for up to 2 years appears to provide no bone benefits in older females. The effects of CR alone on bone measures in older males is unknown. Despite its potential, the effects of CR in older adults with sarcopenia, osteoporosis, osteosarcopenia, sarcopenic obesity, physical frailty, and cachexia remain largely unknown and warrant future long-term clinical trials involving large sample sizes.

## Figures and Tables

**Table 1:** Summary of studies examining creatine and resistance training on muscle outcomes in older adults.

First Author, Year	Population	Supplement Dose	Resistance Training	Duration	Outcomes
Aguiar et al. 2013 [18]	*N* = 18; healthy women; Mean age = 65 y	CR (5 g/day), PLA	RT = 3 x/wk	12 wks	CR ↑ gains in fat-free mass (+3.2%), muscle mass (+2.8%), 1 RM bench press, knee extension, and biceps curl compared to PLA
Alves et al. 2013 [19]	*N* = 47; healthy women, Mean age = 66.8 y (range: 60–80 y)	CR (20 g/day for 5 days, followed by 5 g/day thereafter), PLA with and without RT	RT = 2 x/wk	24 wks	↔1 RM strength compared to RT + PLA
Bemben et al. 2010 and Eliot et al. 2008 [20,21]	*N* = 42; healthy men; age = 48–72 y	CR (5 g/day), PRO (35 g/day), CR + PRO, PLA	RT = 3 x/wk	14 wks	↔lean tissue mass, 1 RM strength
Bermon et al. 1998 [22]	*N* = 32 (16 men, 16 women); healthy; age = 67–80 y	CR (20 g/day for 5 days followed by 3 g/day), PLA	RT = 3 x/wk	7.4 wks (52 days)	↔lower limb muscular volume, 1- and 12-repetitions maxima, and isometric intermittent endurance
Bernat et al. 2019 [23]	*N* = 24 healthy men; age = 59 ± 6 y	CR (0.1 g/kg/day), PLA	High-velocity RT = 2 x/wk	8 wks	↔muscle thickness, physical performance, upper-body muscle strength; CR ↑ leg press strength, total lower body strength
Brose et al. 2003 [24]	*N* = 28 (15 men, 13 women); healthy; age: men = 68.7, women = 70.8 y	CR (5 g/day), PLA	RT = 3 x/wk	14 wks	CR ↑ gains in lean tissue mass and isometric knee extension strength; ↔ type 1, 2 a, 2 x muscle fibre area
Candow et al. 2008 [25]	*N* = 35; healthy men; age = 59–77 y	CR (0.1 g/kg/day), CR + PRO (PRO: 0.3 g/kg/day), PLA	RT = 3 x/wk	10 wks	CR ↑ muscle thickness compared to PLA. CR ↑1 RM bench press ↔ 1 RM leg press
Candow et al. 2015 [26]	*N* = 39 (17 men, 22 women); healthy; age = 50–71 y	CR (0.1 g/kg) before RT, CR (0.1 g/kg) after RT, PLA	RT = 3 x/wk	32 wks	CR after RT ↑ lean tissue mass, 1 RM leg press, 1 RM chest press compared to PLA
Candow et al. 2020 [27]	*N* = 38; healthy men; age = 49–67 y	CR (On training days: 0.05 g/kg before and 0.05 g/kg after exercise) + 0.1 g/kg/day on non-train-ing days (2 equal doses) or PLA	RT = 3 x/wk	12 months	↔lean tissue mass, muscle thickness, or muscle strength
Chilibeck et al. 2015 [28]	*N* = 33; healthy women; Mean age = 57 y	CR (0.1 g/kg/day), PLA	RT = 3 x/wk	52 wks	↔lean tissue mass and muscle thickness gains between groups; ↑ relative bench press strength compared to PLA.
Chrusch et al. 2001 [29]	*N* = 30; healthy men; age = 60–84 y	CR (0.3 g/kg/d for 5 days followed by 0.07 g/kg/day), PLA	RT = 3 x/wk	12 wks	CR ↑ gains in lean tissue mass; CR ↑1 RM leg press, 1 RM knee extension, leg press endurance, and knee extension endurance; ↔ 1 RM bench press or bench press endurance.
Cooke et al. 2014 [30]	*N* = 20; healthy men; age = 55–70 y	CR (20 g/day for 7 days followed by 0.1 g/kg/day on training days)	RT = 3 x/wk	12 wks	↔lean tissue mass, 1 RM bench press, 1 RM leg press
Deacon et al. 2008 [31]	*N* = 80 (50 men, 30 women); COPD; age = 68.2 y	CR (22 g/day for 5 day followed by 3.76 g/day), PLA	RT = 3 x/wk	7 wks	↔lean tissue mass or muscle strength
Eijnde et al. 2003 [32]	*N* = 46; healthy men; age = 55–75 y	CR (5 g/day), PLA	Cardiorespiratory + RT = 2–3 x/wk	26 wks	↔lean tissue mass or isometric maximal strength
Gualano et al. 2011 [33]	*N* = 25 (9 men, 16 women); type 2 diabetes; age = 57 y	CR (5 g/day), PLA	RT = 3 x/wk	12 wks	↔lean tissue mass
Gualano et al. 2014 [34]	*N* = 30; "vulnera-ble" women; Mean age = 65.4 y	CR (20 g/day for 5 days; 5 g/day thereafter), PLA with and without RT	RT = 2 x/wk	24 wks	CR + RT ↑ gains in 1RM bench press and appendicular lean mass compared to PLA + RT
Johannsmeyer et al. 2016 [35]	*N* = 31 (17 men, 14 women); healthy; age = 58 y	CR (0.1 g/kg/day), PLA	RT = 3 x/wk	12 wks	CR ↑ gains in lean tissue mass; ↔ 1RM strength and endurance; CR attenuated magnitude increase in time to complete balance test compared to PLA
Neves et al. 2011 [36]	*N* = 24 (postmen-opausal women with Knee osteo-arthritis); Age = 55–65 y	CR (20 g/day for 1 week, followed by 5 g/day), PLA	RT = 3 x/wk	12 wks	CR ↑ gains in limb lean mass. ↔ 1RM leg press
Pinto et al. 2016 [37]	*N* = 27 (men and women); healthy; age = 60–80 y	CR (5 g/day), PLA	RT = 3 x/wk	12 wks	CR ↑ gains in lean tissue mass; ↔ 10 RM bench press or leg press strength
Smolarek et al. 2020 [38]	*N* = 26 (5 men, 21 women); long-term care residence; age = 68.9 ± 6.8 y	CR (5 g/day), PLA	RT = 2 x/wk	16 wks	CR ↑ dominant and non-dominant handgrip strength

CR = creatine; PRO = protein; RM = repetition maximum; ↑ = significant greater; ↔ no difference between conditions; wk = weeks; y = years; g = grams; kg = kilograms.

**Table 2:** Study characteristics and outcomes of research examining the influence of creatine with a resistance training program on bone.

First Author, Year	Study Population	Intervention	Duration	Outcomes
Brose et al. 2003 [24]	*N* = 28; healthy (15 men, 13 women); age ≥ 65 y (men = 68.7 y, women = 70.8 y)	RCT; CR + RT, PLA + RT. CR = 5 g/day; RT = 3 x/wk	14 wks	↔on osteocalcin
Candow et al. 2008 [25]	*N* = 35; older men (age: 59–77 y)	RCT; CR + PRO + RT; CR + RT, PLA + RT; CR = 0.1 g/kg/day; RT = 3 x/wk	10 wks	CR ↓ NTx
Candow et al. 2019 [5]	*N* = 39; healthy (17 men; 22 women); age ≥ 50 y (mean ~55 y)	RCT; CR-Before + RT, CR-After + RT, PLA + RT; CR = 0.1 g/kg/day; RT = 3 x/wk	8 mths	↔BMD and BMC of the whole-body, limbs, femoral neck, lumbar spine, and total hip
Candow et al. 2020 [27]	*N* = 38; healthy men; age = 49–67 y	RCT; CR + RT, PLA + RT; CR = 0.1 g/kg/day; RT = 3 x/wk	12 mths	↔ BMD and geometry, bone speed of sound; CR ↑ (*p* = 0.06) section modulus of the narrow part of the femoral neck
Chilibeck et al. 2005 [45]	*N* = 29; older men (71 y).	RCT; CR + RT, PLA + RT; CR = 0.3 g/kg/day for 5 days followed by 0.07 g/kg/day for the remaining; RT = 3 x/wk	12 wks	↑ arm BMC greater in the CR group com-pared to PLA; ↔ between groups for whole-body and leg BMD
Chilibeck et al. 2015 [28]	*N* = 33; postmenopausal women; age: 57 ± 6 y	RCT; PLA + RT, CR + RT; CR = 0.1 g/kg/day (0.05 g/kg provided immediately before and 0.05 g/kg after training on training days and with two meals on non-training days); RT = 3 x/wk	12 mths	CR attenuated rate of femoral neck BMD loss compared to PLA and CR ↑ femoral shaft subperiosteal width; ↔ between groups on all other outcome measures
Gualano et al. 2014 [34]	*N* = 60; older vulnerable women (age: 66 y)	RCT; PLA, CR, PLA + RT, CR + RT; CR = 20 g/day for 5 days followed by 5 g/day for the remaining; RT = 2 x/wk	24 wks	↔bone mineral and serum bone markers between groups
Pinto et al. 2016 [37]	*N* = 32; healthy, non-athletic men and women between 60–80 y	RCT; PLA + RT, CR + RT; CR = 5 g/day; RT = 3 x/wk. Muscle groups (i.e., upper and lower body) alternated between training days, 1.5 x/wk per muscle group	12 wks	↔BMD and BMC of all assessed sites between groups

RCT = randomized controlled trial; PLA = placebo; RT = resistance training; CR = creatine; PRO = protein; RM = repetition maximum; NTx = cross-linked N-telopeptides of type I collagen; BMD = bone mineral density; BMC = bone mineral content; ↑ = significant greater; ↔ no difference be-tween conditions; wk = weeks; mth = months; y = years; g = grams; kg = kilograms.

**Table 3:** Studies examining Cr supplementation in a cancer context.

Authors (Year)	Patients	Treatment Modality	Dosage	Protocol Du-RATION	Compliance	Exercise Program	Results	Adverse Effects Related to Supplementation
Jatoi et al. (2017) [98]	263 cancer patients (65 ± 11 yrs.) with weight loss syndrome	210 undergoing concurrent chemotherapy	20 g/day for 5 days then 2 g/day	39 weeks	nr	n/a	↔body weight, appetite, QoL, frailty, grip strength	None reported
Bourgeois et al. (2008) [99]	9 children (7.6 ± 3.8 yrs.) with ALL under-going chemotherapy	Maintenance phase of treatment on the Dann-Farber Cancer Institute protocol 2000–2001	0.1 g/kg/day	2 × 16 weeks separated by 6-week wash-out period.	nr	n/a	↓ BF% Cr, ↑ BF% NH, ↔BMD	None reported
Norman et al. (2006) [100]	31 stage III/IV colorectal cancer patients (65.10 ± 12.55 yrs.) undergoing chemotherapy	*n* = 11: fluorouracil/folic acid (5-FU FA); *n* = 9: fluorouracil/folic acid + oxaliplatin (5-FU FA + O); *n* = 11: fluorouracil/folic acid + irinotecan (5-FU FA + I)	20 g/day for 7 days then 5 g/day	8 weeks	Cr: 84.55 ± 7.77%; PLA: 87.62 ± 5.90%	n/a	↔ weight, capacitance, KE, HR, BCM, BF; ↑ HG 5-FU FA: ↑ phase angle, ECM/BCM ratio	None reported
Lonbro et al. (2013) [101]	30 Head and neck patients treated with radiotherapy	Radiotherapy according to DA-HANCA guidelines (www.da-hanca.dk) + chemotherapy (*n* = 20: cisplatin, 40 mg/m^2^). *N* = 4 received Zalutumumab	5 g/day + 30 g Pro/day	12 weeks	69% ingested all supplementation; 19% missed ≤ 3 supplementations; 12% terminated 4 weeks early	3 days/wk., 3 × 10 total body	↑LBM ProCr group, ns↑PLA ↔ muscle strength **, ↔ Physical function **	No major adverse events reported; 2 participants stopped supplementation 4 weeks early due to muscle cramping and mucus production

ALL: acute lymphoblastic leukemia; BCM: body cell mass; BF%: body fat percentage; BMD: bone mineral density; Cr: creatine supplementation; ECM: extracellular mass; g: gram; HG: hand grip; HF: hip flexion; Kg: kilogram; KE: knee extension; LBM: lean body mass; MF: muscle function; NH: natural history group; PLA: placebo; ProCr: protein + creatine supplementation; n/a: not applicable; nr: not reported; QoL: quality of life; yrs: years old; ↑: increase; ↓: decrease; ↔: no change; ** compared to placebo. (Reproduced with permission from Fairman et al. 2019 [97]).
